# Evaluation of Molecular Serotyping Assays for Shigella flexneri Directly on Stool Samples

**DOI:** 10.1128/JCM.02455-20

**Published:** 2021-01-21

**Authors:** Jie Liu, Suporn Pholwat, Jixian Zhang, Mami Taniuchi, Rashidul Haque, Masud Alam, John Benjamin Ochieng, Jennifer A. Jones, James A. Platts-Mills, Sharon M. Tennant, Eric Houpt

**Affiliations:** a Division of Infectious Diseases and International Health, University of Virginia, Charlottesville, Virginia, USA; b International Centre for Diarrhoeal Disease Research, Bangladesh (icddr,b), Dhaka, Bangladesh; c Kenya Medical Research Institute, Center for Global Health Research (KEMRI-CGHR), Kisumu, Kenya; d Center for Vaccine Development and Global Health, University of Maryland School of Medicine, Baltimore, Maryland, USA; Medical College of Wisconsin

**Keywords:** PCR, *Shigella flexneri*, serotype, stool

## Abstract

Shigella flexneri is prevalent worldwide and is the most common *Shigella* species in many countries. At least 19 S. flexneri serotypes exist, and serotype information is important for epidemiologic and vaccine development purposes. We evaluated the performance of real-time PCR assays for O-antigen modification genes to identify the major serotypes on isolates and direct stool samples. The assays were formulated into two multiplex panels: one panel included *gtrII*, *gtrV*, *gtrX*, *oac*, and *wzx6* to identify S. flexneri serotypes 2a, 2b, 3a, 5a, 5b, 6, and X, and the other panel included *ipaH, gtrI*, *gtrIc*, and *gtrIV* to confirm *Shigella* detection and further identify S. flexneri serotypes 1a, 1b, 1d, 3b, 4a, 4b, 7a, and 7b. We first evaluated 283 *Shigella* isolates, and PCR serotyping demonstrated 97.0% (95% confidence interval, 93.0% to 99.0%) sensitivity and 99.9% (99.9% to 100%) specificity compared to conventional serotyping. The assays then were utilized on direct stool specimens. A quantitative detection algorithm was developed with a validation set of 174 *Shigella* culture-positive stool samples and further tested with a derivation set of 164 samples. The PCR serotyping on stool achieved 93% (89% to 96%) sensitivity and 99% (99% to 100%) specificity compared to serotyping. Most discrepancies were genotypic-phenotypic discordance, not genotypic failure. These real-time PCR assays provide an efficient and novel tool for S. flexneri serotype identification.

## Abstract (precis)

Shigella flexneri is prevalent worldwide and is the most common *Shigella* species in many countries. At least 19 S. flexneri serotypes exist, and serotype information is important for epidemiologic and vaccine development purposes.

## INTRODUCTION

Shigellosis is a leading cause of childhood diarrhea worldwide, second only to rotavirus (1). The use of real-time PCR diagnostics has substantially improved the detection of *Shigella* such that an even greater proportion of moderate and severe diarrhea burden is now attributable to this pathogen (1–5). Because of this high burden of disease and increasing antibiotic resistance, *Shigella* vaccine development is under active investigation.

*Shigella* encompasses four species: S. flexneri, S. sonnei, S. dysenteriae, and S. boydii. S. flexneri is the predominant species, followed by S. sonnei (6, 7). Each is composed of different serotypes, which are determined by the structure of lipopolysaccharide O-antigen repeats. Because most *Shigella* vaccine candidates target serotype-specific antibody responses (8), identification of circulating S. flexneri serotypes is increasingly relevant. S. flexneri consists of two distinct lineages. One lineage consists of serotype 6, which possesses a unique O-antigen tetrasaccharide repeat (9). The other lineage consists of all other S. flexneri serotypes (1 to 5, 7, X, and Y) (10). S. sonnei is represented by a single O-antigen serotype.

The standard methods for serotype identification involve agglutination with diagnostic antisera. This procedure is time-consuming and costly, requiring up to 10 separate agglutination tests using antisera for type antigens I, II, III, IV, V, and VI, antisera for group antigens 3 and 4, 7 and 8, and 6, and the monoclonal antibody MASF1c for serotype 1c (11, 12). Additionally, results require visual assessment of agglutination reactions and a complex interpretation scheme that can be ambiguous (13). It is also found that there can be differences in results obtained using antisera from different suppliers.

Since *Shigella* serotypes are determined by the glucosyl and/or *O*-acetyl modifications to the O-antigen tetrasaccharide repeat, which are conferred by *gtr* and/or *oac* genes, respectively (6, 14), detection of these genes can also predict serotype. These genes are encoded by different prophage genomes incorporated into the host chromosome (15). A blind multicenter evaluation found that a multiplex PCR-gel electrophoresis assay for these genes performed on isolates delivered good agreement with the conventional serotyping methodology (16).

In the present study, we sought to expand on this prior work by developing a multiplex real-time PCR assay targeting specific *gtr* and *oac* O-antigen modification genes to serotype S. flexneri on not only isolates but also direct stool specimens. Except for the rare serotype Y, the most common S. flexneri serotypes were amenable to this approach. This multiplex PCR assay provides a rapid and specific method for molecular serotyping of S. flexneri and can be performed on direct stool to enhance *Shigella* epidemiology.

## MATERIALS AND METHODS

### Specimens.

**FIG 1:**
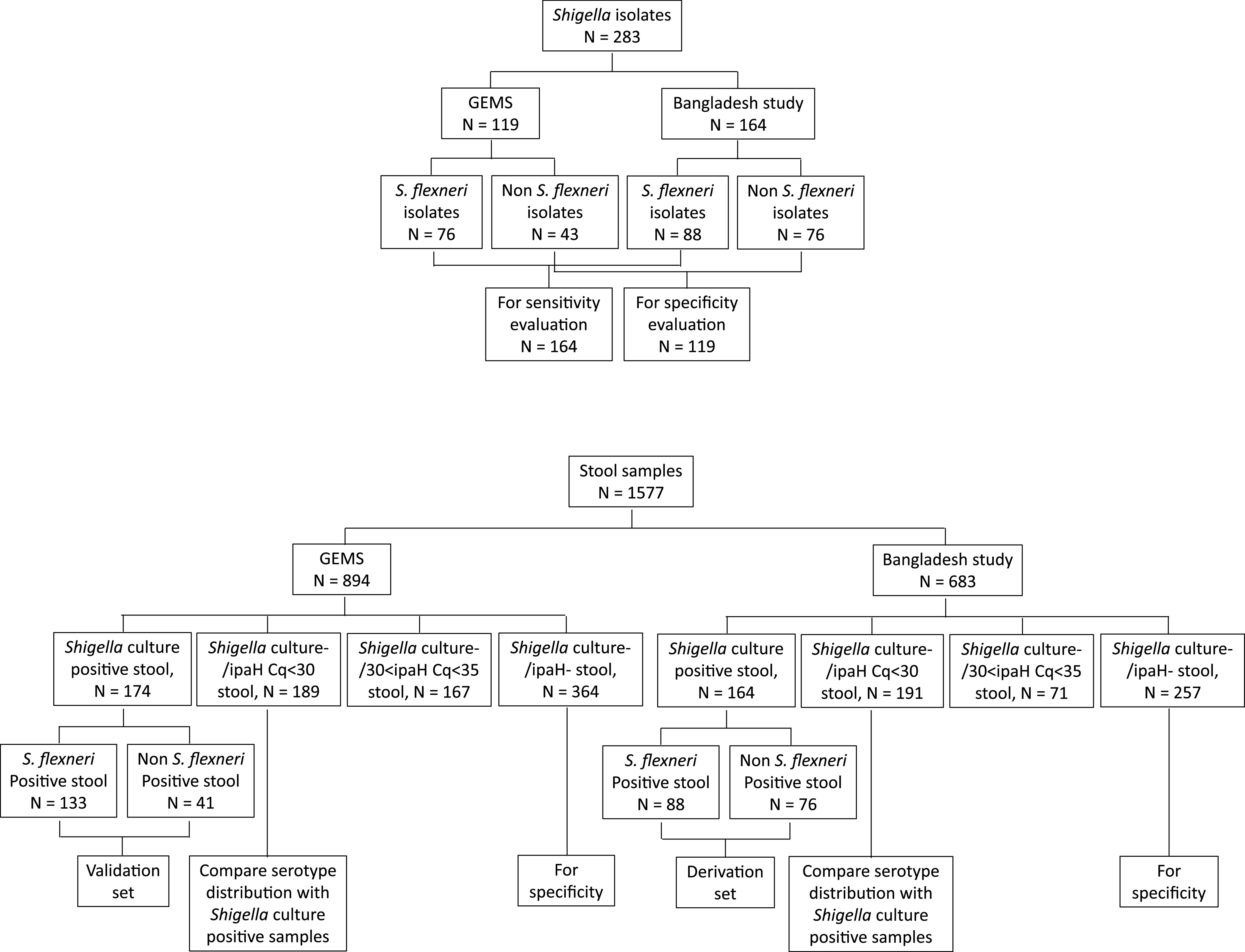
Experimental scheme demonstrated with flow charts. Specimens collected in GEMS and the Bangladesh *Shigella* Outcome Study were used in the current work.

Figure 1 shows the *Shigella*-positive isolates and stool samples used in the current work. A total of 119 isolates of *Shigella* species and 894 stool samples were collected through the Global Enteric Multicenter Study (GEMS) (1, 3). The isolates were chosen to provide a mixture of serotypes and were shipped from the University of Maryland School of Medicine to the University of Virginia. The GEMS microbiological methods were described previously (6, 17). Briefly, stool samples were plated onto MacConkey and xylose lysine deoxycholate agar, and then suspicious colonies underwent biochemical tests for species identification. There were 174 stool samples from which *Shigella* had been cultured and serotyped during GEMS. Another 720 *Shigella* culture-negative stool samples were randomly selected from the study and also tested. In addition, a recent observational *Shigella* study from the International Centre for Diarrheal Diseases and Research, Bangladesh (icddr,b) (18), provided all of the *Shigella* isolates (*n* = 164) and stools (*n* = 683). These isolates were serotyped and stools tested at the University of Virginia. The University of Virginia, icddr,b, and GEMS sites Institutional Review Boards approved this work.

### Serotyping of *Shigella* isolates.

*Shigella* isolates from GEMS were serotyped with polyvalent group A, B, C, and D antisera and shipped to the GEMS Reference Laboratory at the Center for Vaccine Development and Global Health for confirmation. One-third of isolates were further sent to the Centers for Disease Control and Prevention (CDC) for serotype confirmation, which yielded >99% concordance. S. flexneri serotypes from the Bangladesh study were identified using monovalent antisera from Denka Seiken (Japan) by following the manufacturer’s instructions, with confirmation of results at the CDC.

### Nucleic acid extraction.

One milliliter of overnight culture in tryptic soy broth (14 to 16 h at 37°C with shaking) of each isolate was pelleted and then resuspended in 200 μl of TE buffer (10 mM Tris-HCl, 1 mM EDTA, pH 8.0) and boiled for 20 min. The supernatant was collected after a 5-min spin at 12,000 × *g* and used directly for PCR. Stool samples were transported on dry ice and stored at –80°C until testing. Nucleic acid was extracted from 200 mg of stool sample using the QIAamp Fast stool DNA minikit (Qiagen, Hilden, Germany) with pretreatments and spiked with external controls as described previously (19). One extraction blank was included per batch of extraction to rule out laboratory contamination.

### PCR design.

Real-time PCR primers and probes were adapted or modified from Gentle et al. (20) as shown in Table 1. *In silico* specificity was screened and confirmed with BLAST searches. Two multiplex real-time PCR panels were formulated, including the *ipaH* assay, to confirm *Shigella* detection. Primers were synthesized by Integrated DNA Technologies (IDT; Coralville, IA), and probes were synthesized by IDT and BioSearch (Novato, CA). The assays were also compartmented into a customized TaqMan array card (TAC; Life Technologies, Carlsbad, CA) along with other enteric pathogens as described previously (21), whereby TaqMan MGB probes were utilized.

**TABLE 1:** Primer and probe sequences for identification of various Shigella flexneri serotypes^*a*^

Target	Accession no.	Sequence	Concn (nM)	Amplicon length (bp)	Reactive S. flexneri serotype(s)
Panel I					
*gtrII*	AY900451	CAAACGACTCAGGAAATATGC	150	104	2a, 2b
		AATTCATAAATGCAACCATCCT	150		
		FAM-CTCCATGAGCGCAGACACTTTTG	75		
*gtrV*	CP000266	TTATTAATGTCATCGTCCATCC	400	112	5a, 5b
		CCACTCCCAGATTACGG	400		
		Quasar670-GCAGGGTTGAACTTGAAAGAATACGAT	200		
*gtrX*	L05001	AGCACCACATCAAAAATCTTC	500	106	1d, 2b, 3a, 5b, X
		CATACAATGATAAATACCAGTGAGCATT	500		
		HEX-TATATTTAATTTGCATGCCCGGGC	250		
*wzx6*	EU294165	GAGCGATCATTTCAACTTCA	500	122	6
		TACAACATGATTCGCGTTAATGT*	500		
		Quasar 705-CGGTAATTCTAACTATATTGGGCTTG*	250		
*oac*	X59553	GCATAAGAGCAACTGCTTTG	400	73	1b, 3a, 3b, 4b, 5a, 5b, 7b
		CGCGTAGTGGTGACTG	400		
		Texas Red-ACGGCAAGGCTTGTGGCA	200		
Panel II					
*gtrI*	AF139596	AAATATGCCTCCATACAATTG	500	133	1a, 1b, 1d, 7a, 7b
		AGCATATGTATTAAACAATCAGCA	500		
		FAM-GCTGTTAGCAACATCCGGTTCAAC	250		
*gtrIc*	FJ905303	ACCTTAGGTTCAAATGGGTTAC	400	132	7a, 7b
		GAAATAGCCGTCTCTCGAATA	400		
		Quasar 670-TGTTTTCACATTTAGTATTCCAAC*	200		
*gtrIV*	AF288197	TCTCACATGATGGCACATTA	400	143	4a, 4b
		CCTAAGATCAAATGTGTGTGTGA	400		
		HEX-TTTATACCCTGAAGGAAAATTTCAG*	200		
*ipaH*	M76445	CCTTTTCCGCGTTCCTTGA	125	64	All *Shigella* species and serotypes
		CGGAATCCGGAGGTATTGC	125		
		Quasar 705-CGCCTTTCCGATACCGTCTCTGCA	62.5		

a Assays were adapted from Gentle et al. (20), with some modifications marked with asterisks. *gtr* genes encode serotype-specific glucosyltransferases; *oac* encodes an *O*-acetyltransferase that mediates the addition of an acetyl group to a specific sugar in the backbone structure; *wzx6* is the O-antigen synthesis gene specific to S. flexneri serotype 6.

### PCR conditions.

Each 20-μl multiplex PCR included 10 μl of 2× AgPath ID one-step reverse transcription-PCR (RT-PCR) buffer (Life Technologies, Carlsbad, CA), 0.8 μl of enzyme mix, 1 μl of cell lysate or stool nucleic acid extract, and primers and probes at the concentrations indicated in Table 1. Cycling conditions included 20 min of reverse transcription at 45°C (to be consistent with the cycling conditions on TAC), 15 min of initial denaturation at 95°C, and 40 cycles of 15 s at 95°C and 1 min at 60°C. A pooled positive control at a concentration of 10^6^ copies/ml (mixed genomic DNA purified from Shigella flexneri isolates covering all assay targets) and a no template negative control were included on each plate. The stool samples collected from the Bangladesh *Shigella* study were tested on TaqMan array cards under the conditions previously described (1). Briefly, 20 μl of extracted nucleic acid was mixed with AgPath ID one-step RT-PCR reagents in a 100-μl volume and loaded onto the TaqMan array card. The RT-PCR cycling conditions were identical to those described above. Multiplex PCR panels and TaqMan Array cards were performed on CFX (Bio-Rad, Hercules, CA) and a ViiA 7 real-time PCR system (Life Technologies, Carlsbad, CA), and the results were analyzed with CFX Maestro software v1.1 (Bio-Rad) and QuantStudio real-time PCR software v1.3 (Life Technologies), respectively. Quantification cycles (Cqs) were determined with the baseline threshold setting.

### Assay validation.

Linearity, precision, lower limit of detection, and specificity were tested for both panels. Linearity, precision, and lower limit of detection were measured using the strains listed in Table S1 in the supplemental material. Linearity was tested with a 10-fold serial dilution of the corresponding material in three replicates. For the limit of detection, 200 mg of stool from healthy donors was spiked with 10^3^ to 10^5^ CFU of cultured isolates and then extracted and assayed. The limit of detection was defined as the lowest concentration at which the target could be detected in all 10 spiked samples. Intra-assay precision was tested with 10 repeats within one run of a sample spiked with 10^5^ CFU, and interassay precision was tested with 10 identically spiked samples with 10^5^ CFU that were extracted and assayed over 5 days. Specificity was tested against a panel of enteropathogens listed in Table S2.

### Assay performance.

The real-time PCR assays, in the format of either multiplex or TaqMan array card, were performed on the *Shigella* isolates and stool samples. The serotype results were compared between real-time PCR and conventional agglutination. Discrepant samples were amplified with the relevant PCR assays and subjected to amplicon sequencing (22).

### Statistics.

Correlation was tested by regression analysis using the analysis of variance (ANOVA) test. Cq cutoffs were determined with receiver operating characteristic (ROC) analysis. Two-tailed *P* values were calculated, and values of <0.05 were considered statistically significant. All analyses were performed using IBM SPSS Statistics version 26.

## RESULTS

### Assay performance on *Shigella* isolates.

**FIG 2:**
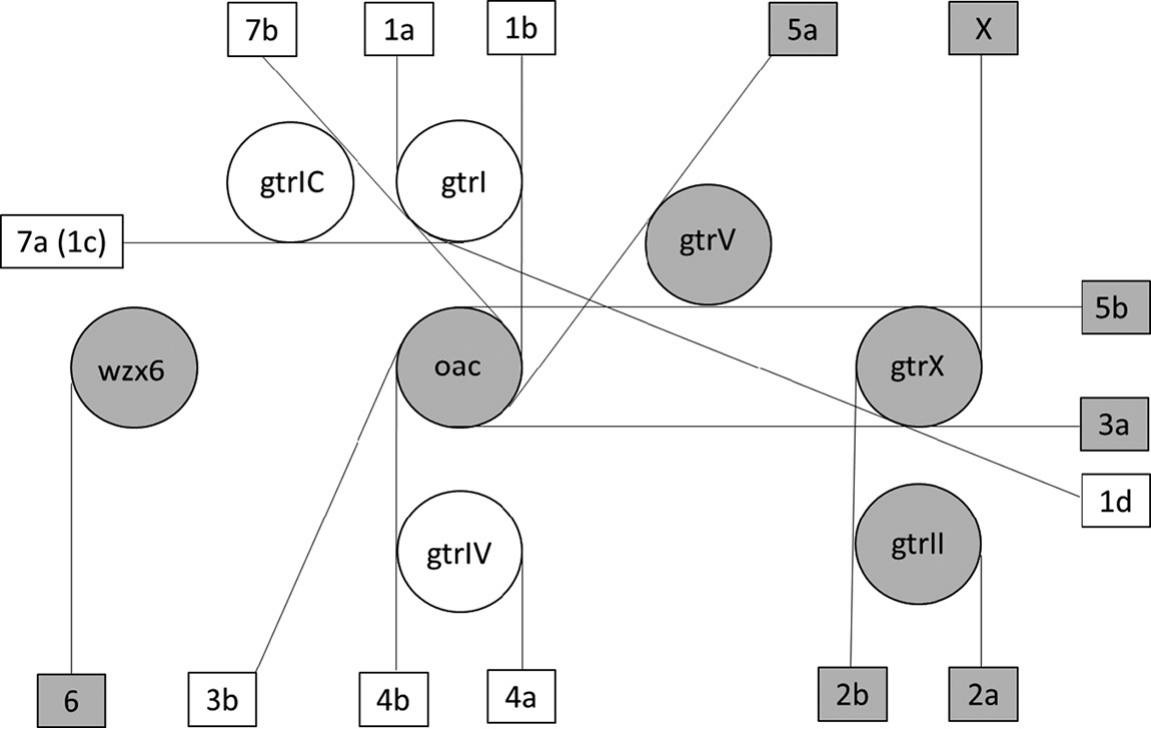
Algorithm for Shigella flexneri serotype identification with real-time PCR assays. Each circle indicates one PCR target and each rectangle indicates one Shigella flexneri serotype, while each straight line links the required PCR targets to the relevant serotype, i.e., the PCR targets in the circles touching a straight line all have to be present to call the serotype at the end of that line. The shaded symbols indicate the first multiplex panel, and the white symbols indicate the second panel.

We collected 283 *Shigella* isolates, including 164 S. flexneri isolates, that constituted all major serotypes except X, 1d, and 7b. Because the interrogated gene targets can be found in one or more serotypes, an interpretation algorithm similar to that for antisera was used to define serotype from the PCR result (Fig. 2). This PCR assay algorithm correctly identified the serotypes identified by conventional methods with 97.0% sensitivity (95% confidence interval [CI], 93.0% to 99.0%; 159/164; Table 2) and 99.9% specificity (99.9% to 100.0%; 3,510/3,515). Analytical performance of the multiplex assays is shown in Table S1. The PCR efficiencies were 86 to 95%, and linearities (*R*^2^) were 0.980 to 0.994, indicating that the assays were quantitative. The lower limit of detection was between 10^4^ and 5 × 10^4^ CFU per gram of stool, depending on the target. No cross-reactivity with the PCR assays was observed against a large panel of enteropathogens (Table S2) as well as with the no-template controls.

**TABLE 2:** *Shigella* isolates used for validation in this study

Serotype/species	No. isolates tested (no. of correct S. flexneri serotype identifications, %)
S. flexneri 2a	84 (84, 100)
S. flexneri 3a	21 (18, 86)^*a*^
S. flexneri 6	15 (15, 100)
S. flexneri 1a	3 (1, 33)^*b*^
S. flexneri 1b	15 (15, 100)
S. flexneri 2b	10 (10, 100)
S. flexneri 3b	1 (1, 100)
S. flexneri 4a	7 (7, 100)
S. flexneri 4b	1 (1, 100)
S. flexneri 5a	3 (3, 100)
S. flexneri 5b	1 (1, 100)
S. flexneri 7a	3 (3, 100)
S. sonnei	86 (0)
S. boydii	17 (0)
S. dysenteriae	16 (0)
Total	283 (159/164, 97)

a The 3 discordant samples were identified as serotype 5b (*oac*, *gtrX*, and *gtrV*) instead of 3a (*oac* and *gtrX* only).

b The 2 discordant samples were identified as serotype 7a (*gtrI* and *gtrIc*) instead of 1a (*gtrI* only).

### Validation of assay performance on stool samples culture positive for *Shigella*.

A total of 174 *Shigella* culture-positive stool samples from the GEMS study whose cultured isolates previously underwent speciation and conventional serotyping were then tested with these PCR assays. Two of the stool specimens did not yield amplification of any of the *Shigella* PCR targets, including *ipaH*, and were excluded from analysis. Specificity testing showed that no S. flexneri serotype was identified for all 41 stool samples that were culture positive for S. sonnei, S. boydii, or S. dysenteriae. Unlike the PCR detections on isolates that yielded Cq between 18 and 24 (20), the Cq values on stool samples ranged from below 20 to the high thirties. On stool samples, the correlation of serotype-specific Cqs with *ipaH* Cqs (see Fig. S1A in the supplemental material) was mostly above 0.9 (*R*^2^), although the average Cq values of the serotype targets were 4.8 ± 1.1 higher than those of *ipaH*. When two gene targets were required to discern a serotype, the Cqs of the two serotyping targets were within 0.9 ± 0.8 Cq of each other (0.9 ± 0.4, 0.3 ± 0.9, 1.5 ± 0.3, and 0.4 ± 0.3 for serotype 1b, 2b, 3a, and 7a, respectively; Fig. S1B). ROC analysis showed that 95.1% of the *ipaH*-positive stools with *ipaH* Cq of <30.4 were serotypeable.

Therefore, we devised the following quantitative algorithm to determine serotype on direct stool specimens: (i) require *ipaH* Cq of <30, (ii) require Cq of serotyping target fall within 5 Cq of the *ipaH* Cq, and (iii) if two or more targets are required to determine the serotype, the Cq difference must fall within 2 Cq of each other. Utilizing these criteria, 93% of the 133 S. flexneri isolates from GEMS (out of 174 total *Shigella* isolates) were correctly predicted by stool PCR. The discrepancies included one stool sample with *Shigella* conventionally serotyped as 1a and identified molecularly as 1b because of the additional detection of *oac*; one 1b identified as 6 because of the detection of *wzx6*; one 3a identified as 5b because of the additional detection of *gtrV*; one 3b identified as 5a because of the additional detection of *gtrV*; three 3b identified as 1b because of the additional detection of *gtrI*; and two 5b identified as 2b because of the additional detection of *gtrII* without *oac*.

### Derivation of the quantitative algorithm for PCR serotyping directly on stool samples.

The above-described criteria were further applied to a derivation set of 164 *Shigella* culture-positive stool samples collected from the Bangladesh study. This yielded 93% (85.8% to 97.5%) sensitivity on 88 S. flexneri samples and 99% (97.4% to 99.4%) specificity. The discrepancies were the following: two 3a identified as 5b because of the additional detection of *gtrV*; two 1a identified as 7a because of the additional detection of *gtrIc*; one serotype 6 identified as mixed infection of 3a and 6, with 3a at slightly higher quantity; and one 7a identified as 2a. The serotype results for the combined collection of 221 S. flexneri culture-positive stool samples (out of 338 *Shigella* species culture-positive samples) from both studies are summarized in Table 3. To assess these discrepancies from both studies, we performed confirmatory PCRs using the primers of Sun et al. (22) and amplicon sequencing, and the presence or absence of all above-mentioned PCR targets was confirmed, except for one 2a detection. Therefore, against a gold standard of serotyping with sequencing of any discrepancies, the overall sensitivity and specificity of the PCR approach were both 99% (CI, 97.5% to 100% for sensitivity, 99.7% to 100% for specificity). Detection of a secondary serotype of lower quantity was identified in two samples from GEMS (1.5%, 2/133), for example, one sample had detection of *ipaH* Cq of 18.0, *gtrI* Cq of 23.3, *gtrII* Cq of 27.2, and *gtrX* Cq of 27.3, consistent with primary S. flexneri 1a plus less abundant S. flexneri 2b. The detection of these extra targets was also confirmed by amplicon sequencing.

**TABLE 3:** Identification of S. flexneri serotype on direct stool samples that were culture positive/*ipaH* positive and were previously serotyped using conventional methods^*a*^

Serotype	No. culture positive	No. detected by PCR (% sensitivity, CI)	No. of other serotypes detected (% specificity, CI)	Sequencing confirmation^*b*^	Corrected % sensitivity/% specificity
*S. flexneri* 2a	93	93 (100, 96–100)	1^*c*^ (99, 96–100)		100, 99
*S. flexneri* 3a	25	22 (88, 69–97)	1 (99, 97–100)	3 (*gtrV*, 5b)	100, 100
*S. flexneri* 6	18	17 (94, 73–100)	1^*c*^ (99, 97–100)	3a, 6 (*oac, gtrX*)	100, 99
*S. flexneri* 1a	6	3 (50, 12–88)	0 (100, 98–100)	1 (*oac*, 1b), 2 (*gtrIc*, 7a)	100, 100
*S. flexneri* 1b	19	18 (95, 74–100)	4 (97, 95–99)	1 (*wzx6*, 6)	100, 100
*S. flexneri* 2b	20	20 (100, 83–100)	2 (99, 96–100)		100, 100
*S. flexneri* 3b	4	0 (0, 0–60)	0 (100, 98–100)	1 (*gtrV*, 5a), 3 (*gtrI*, 1b)	—, 100
*S. flexneri* 4a	15	15 (100, 78–100)	0 (100, 98–100)		100, 100
*S. flexneri* 5a	0		(99, 98–100)		—, 100
*S. flexneri* 5b	2	0 (0, 0–84)	(99, 97–100)	2 (*gtrII*, 2b)	—, 100
*S. flexneri* X	9	9 (100, 66–100)	0 (100, 98–100)		100, 100
*S. flexneri* 7a	10	9 (90, 56–100)	2 (99, 97–100)	2a	90, 100
All	221	206/221 (93, 89–96)	13/2,431 (99, 99–100)		220/221 (99), 2,429/2,431 (99)

a Sensitivity and specificity were calculated compared to conventional serotyping. Corrected sensitivity and specificity then were recalculated, taking into account the sequencing confirmation results.

b This column shows the PCR results that were discrepant from culture. These samples were tested with long amplicon PCR followed by Sanger sequencing. All detections were confirmed except for 2a.

c All additional detections except for these two were confirmed with long amplicon PCR followed by Sanger sequencing.

### Assay performance on stool samples culture negative for *Shigella*.

**FIG 3:**
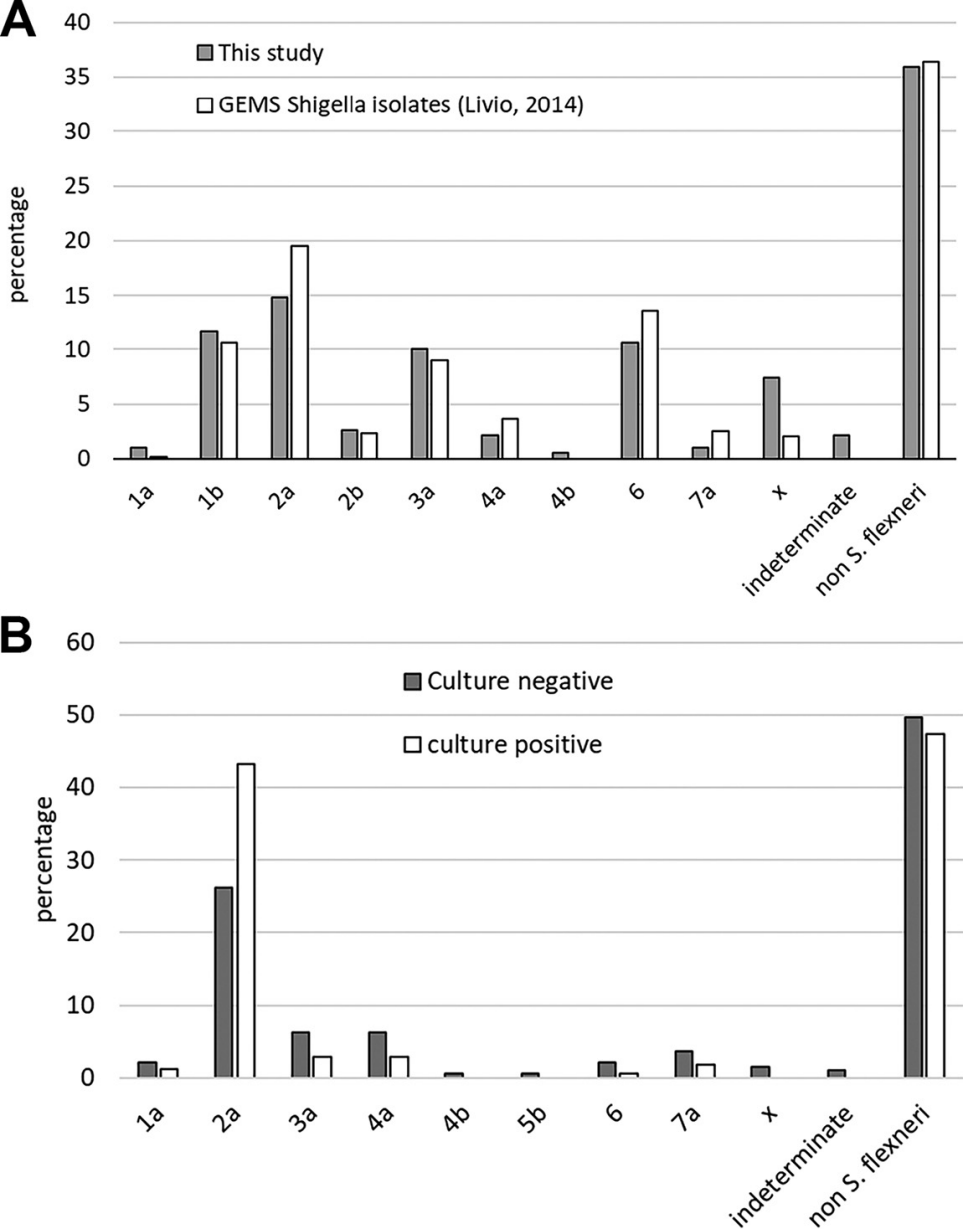
Distribution of S. flexneri serotypes in culture-negative/PCR (*ipaH*)-positive stool samples. (A) Stools from the GEMS (*N* = 189) were serotyped and compared with the previously published serotypes from the cultured isolates. (B) Stools from the Bangladesh *Shigella* study (*N* = 191) were serotyped and compared with the serotypes from the cultured isolates (6). The serotype could not be discerned for 6 stool samples (4 from GEMS study), because multiple serotype targets were PCR positive at similar quantities.

We next performed the multiplex PCR assays on 189 *Shigella* culture-negative but *ipaH* PCR-positive (Cq < 30) samples from the Mali, Mozambique, and India GEMS sites. Figure 3A shows the distribution of S. flexneri serotypes in 63% of these stools, which was comparable to the previously published conventional serotypes from those 3 sites (6). A serotype could not be ascribed in 4 samples because multiple serotype gene targets were present with similar quantities. Similarly, we performed the PCR assays using the TAC format on the 191 samples from the Bangladesh study that were *Shigella* culture negative but *ipaH* PCR positive. Of these samples, 50% yielded an S. flexneri serotype based on the PCR algorithm, again with distribution similar to that of the conventional serotypes of the *Shigella* culture-positive samples (Fig. 3B). We also examined the culture-negative/*ipaH*-negative samples from both studies to further evaluate the specificity. Of these, 93% (576/621) were negative for all *Shigella* serotyping targets, and of the 45 samples positive for one or more serotype targets, all yielded a Cq above 28.

## DISCUSSION

In this work, we designed and evaluated a molecular serotyping method for S. flexneri for use on isolates or direct stool specimens. Eight real-time PCR assays were included targeting the O-antigen glucosyl transferase genes (*gtrI*, *gtrIc*, *gtrII*, *gtrIV*, *gtrV*, and *gtrX*), *oac*, and *wzx6*. These assays can be formulated into two multiplex PCR panels or used on the TaqMan array card.

On isolates, qualitative detection of these genes enabled identification of the *Shigella* serotype, including 1a, 1b, 2a, 2b, 3a, 3b, 4a, 4b, 5a, 5b, 6, 7a (1c), and X, with excellent sensitivity and specificity (>97%). On stool specimens, a quantitative interpretation scheme was devised to enable the identification of the stool’s S. flexneri serotype. For some serotypes, such as 1a, 2a, 3b, 4a, 6, and X, a single gene target is detected. For the serotypes that require two targets, we noted a close quantitative relationship between these two targets. The quantity of serotype targets was consistently about 5 Cq units higher than that of the pan-*Shigella* target *ipaH*, which is unsurprising, since the latter is present in multiple copies on both chromosome and plasmid. These quantitative features formed the basis for our algorithm for determining the S. flexneri serotype on stool specimens, which yielded 93% sensitivity and 99% specificity compared to conventional serotyping results. Most discrepancies showed that the molecular results were supported by independent PCRs and sequencing, indicating they were genotypic-phenotypic discordance, not genotypic deficiencies. Such genotypic-phenotypic discordance has been reported by others previously (16, 20) and may relate to insertions, deletions, or mutations in O-antigen synthesis or modification genes. For example, insertion of *oac* 804:+1T could render an isolate phenotypically 1a but genotypically 1b (20). Likewise, deletion in *gtrV* 646:-1T could render an isolate phenotypically 3b but genotypically 5a (20). Of note, mixed S. flexneri serotype infection appeared to exist but was uncommon (6%; 26 out of a total of 436 S. flexneri serotyped stool samples), and usually one serotype predominated. Assuming proportions of S. flexneri in *ipaH* PCR-positive samples similar to those of *Shigella* culture positives, i.e., 63% in GEMS and 54% in the Bangladesh study, it is estimated that 97.7% (303/310) of S. flexneri positives could be serotyped.

Given that molecular detection is severalfold more sensitive for detecting *Shigella* diarrhea than *Shigella* culture, we need sensitive molecular tools to detect *Shigella* serotypes in culture-negative specimens. In several large multisite studies, we previously determined that the diarrhea-associated *Shigella* quantity is approximately 10^7^ or more copies of *ipaH* gene per gram of stool (1, 4). This *Shigella* quantity is equivalent to an *ipaH* Cq of approximately 26 in a multiplex PCR or 29 with TAC. Therefore, the use of an *ipaH* Cq of <30 for our interpretation algorithm allows good clinical specificity. While there is no cultured isolate to use as a gold standard in *ipaH*-positive/culture-negative stools, it was reassuring to note that the serotype of these *ipaH*-positive/culture-negative stools mirrored the serotypes of the culture-positive stools from two large independent studies.

This work had some limitations. First, the number of positive samples for some serotypes was small, in particular the rare serotypes 1d and 7b, so further evaluation is needed for these serotypes. Next, the quantitative relationship between serotype targets, and between serotype targets and *ipaH*, may vary with different PCR platforms. While our criteria worked using these multiplex PCRs and TAC, for a different molecular platform a validation set of samples should be evaluated prior to implementation. Serotype Y, which consists of only the basic polysaccharide O-antigen without glucosylation or acetylation, cannot be identified with these assays. Likewise, serotype Xv and 4av cannot be distinguished from X and 4a, respectively (14–16).

Based on the cross protection with shared group- and type-specific antigens (23), a multivalent vaccine including O-antigens of S. sonnei, S. flexneri 2a, S. flexneri 3a, and S. flexneri 6 has been proposed. Such a vaccine would be expected to provide direct coverage against approximately 64% of the strains in the large GEMS study (6), with cross protection against approximately 88% of strains belonging to other serotypes. Different serotype distributions were found in the two studies we tested here, with much more 2a in the recent Bangladesh study. This highlights the importance of performing *Shigella* serotyping in several geographic areas and/or at different times to better understand circulating types to inform vaccine development. Long-term surveillance of the serotypes before and after vaccine administration will also be important.

## Supplementary Material

Supplemental file 1
