# Supplementary material for: Evaluation of Molecular Serotyping Assays for Shigella flexneri Directly on Stool Samples
Source: J Clin Microbiol. 2021 Jan 21;59(2):e02455-20. doi: 10.1128/JCM.02455-20 (PMC8111134; doi:10.1128/JCM.02455-20)

Supplemental Table S1. Analytical performance of *Shigella* serotyping assays.

Panel	Target	strain	PCR efficiency (%)	Linearity R^2	Precision (%)		Limit of detection (CFU/g stool)
					Intra-assay	Inter-assay	
I	*gtrII*	*S. flexneri* 2a	91	0.990	1.5	4.3	5×10^4
	gtrV	*S. flexneri* 5a	90	0.993	2.2	3.8	5×10^4
	gtrX	*S. flexneri* 2b	89	0.987	2.1	2.6	5×10^4
	oac	*S. flexneri* 3a	88	0.980	3.2	3.2	5×10^4
	wzx6	*S. flexneri* 6	95	0.991	3.1	4.3	5×10^4
II	*gtrI*	*S. flexneri* 1a	92	0.992	2.1	3.6	5×10^4
	gtrIc	*S. flexneri* 7a*	88	0.989	2.4	3.8	5×10^4
	gtrIV	*S. flexneri* 4a	90	0.994	3.5	4.1	5×10^4
	ipaH	*S. flexneri* 2a	86	0.986	3.8	6.3	10^4

* using synthetic nucleic acid

Supplemental Table S2. Enteropathogen specificity testing. Listed in the table were the pathogens for which genomic materials were available. Stool samples that were positive for astrovirus, norovirus GI, norovirus GII, sapovirus, *E. bieneusi*, were also tested.

	**Species**
Bacteria	EAEC
	EPEC
	ETEC
	STEC
	EIEC
	Aeromonas hydrophila
	Bacteroides fragilis
	Clostridium difficile
	Campylobacter jejuni
	Campylobacter coli
	Campylobacter hyointestinalis
	Campylobacter upsalensis
	H. pylori
	Salmonella enterica
	Vibrio cholerae
	Vibrio parahaemolyticus
	Yersinia enterocolitica
Virus	Adenovirus 5
	Adenovirus 40
	Adenovirus 41
	Cytomegalovirus
	Rotavirus
Parasite	*Blastocytis hominis*
	Cryptosporidium hominis
	Cryptosporidium parvum
	E. histolytica
	E. dispar
	E. intestinalis
	Giardia lamblia
	Schistosoma mansoni

Figure S1. A. Correlation of Cqs between *S. flexneri* serotype targets and *ipaH* on stool samples. For the serotypes that required two gene targets, the two targets are plotted with blue and orange symbols, respectively.

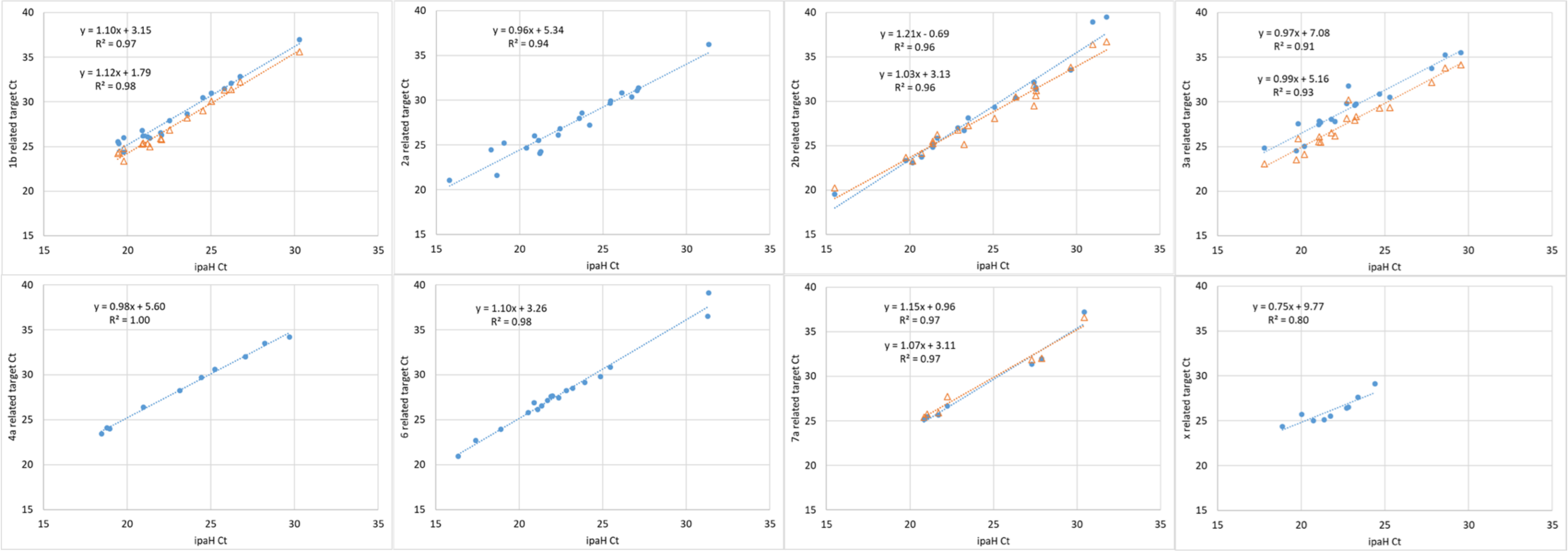

Figure S1. B. Correlation of Cqs between *S. flexneri* serotype target pairs on stool samples.

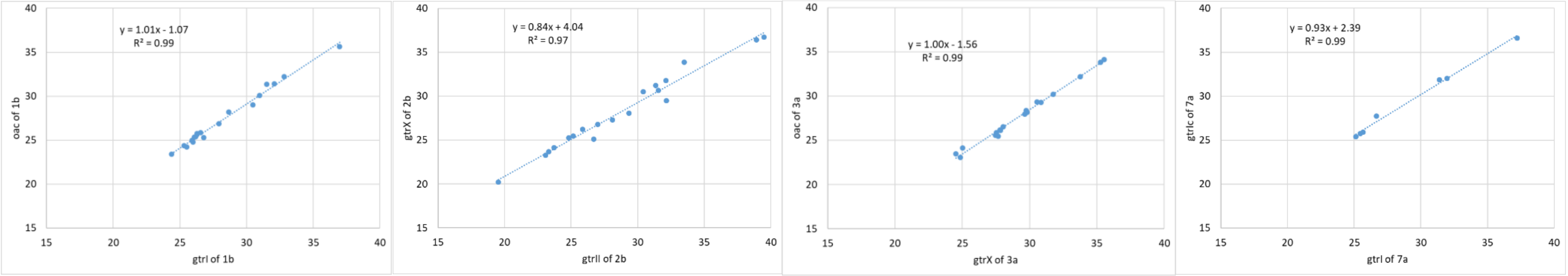

Supplement: Supplemental file 1 [file JCM.02455-20-s0001.pdf]
